# A Gamma Interferon Independent Mechanism of CD4 T Cell Mediated Control of *M. tuberculosis* Infection *in vivo*

**DOI:** 10.1371/journal.ppat.1002052

**Published:** 2011-05-19

**Authors:** Alena M. Gallegos, Jeroen W. J. van Heijst, Miriam Samstein, Xiaodi Su, Eric G. Pamer, Michael S. Glickman

**Affiliations:** 1 NIH/NIAID Laboratory of Parasitic Diseases, Bethesda, Maryland, United States of America; 2 Immunology Program, Infectious Disease Service, Memorial Sloan-Kettering Cancer Center, New York, New York, United States of America; 3 Program in Immunology and Microbial Pathogenesis, Weill Graduate School of Medical Sciences, New York, New York, United States of America; University of Washington, United States of America

## Abstract

CD4 T cell deficiency or defective IFNγ signaling render humans and mice highly susceptible to *Mycobacterium tuberculosis* (Mtb) infection. The prevailing model is that Th1 CD4 T cells produce IFNγ to activate bactericidal effector mechanisms of infected macrophages. Here we test this model by directly interrogating the effector functions of Th1 CD4 T cells required to control Mtb in vivo. While Th1 CD4 T cells specific for the Mtb antigen ESAT-6 restrict in vivo Mtb growth, this inhibition is independent of IFNγ or TNF and does not require the perforin or FAS effector pathways. Adoptive transfer of Th17 CD4 T cells specific for ESAT-6 partially inhibited Mtb growth while Th2 CD4 T cells were largely ineffective. These results imply a previously unrecognized IFNγ/TNF independent pathway that efficiently controls Mtb and suggest that optimization of this alternative effector function may provide new therapeutic avenues to combat Mtb through vaccination.

## Author Summary

*Mycobacterium tuberculosis* (Mtb) is an inhaled pathogen that primarily infects the lungs and causes the disease, tuberculosis. Recent WHO statistics show that more than 2 billion people are infected with Mtb, of these over 1 million people die every year. Researchers over the last several decades have tried to determine how our immune system fights Mtb infection. It is known that CD4 T cells, and the pro-inflammatory cytokine, IFNγ, are required to control Mtb infection in humans and in mice. Based on these observations, it is commonly assumed that vaccines that maximize IFNγ-producing Mtb-specific CD4 T cell numbers will be the most effective. For the first time, we tested this idea directly and our results led us to the unexpected finding that Mtb specific CD4 T cells do not require IFNγ in order to protect mice from Mtb infection. Our results challenge the model that optimization of IFNγ-producing CD4 T cells will optimize vaccine induced protection against *M. tuberculosis*.

## Introduction

IFNγ is essential for defense against Mtb infection, as revealed by experimental studies using knockout mice and the unusually severe mycobacterial infections in patients with defects in the IFNγ or IL-12 signaling pathways [1], [2], [3], [4]. The role of CD4 T cells in defense against Mtb infection has been inferred from the increased reactivation of latent Mtb infections in CD4 T cell deficient patients following HIV infection and severe tuberculosis observed in CD4 T cell-deficient mice [3], [5]. These clinical and experimental findings have led to a widely accepted model positing that the critical immunologic mechanism of anti-mycobacterial immunity involves CD4 T cells that secrete IFNγ to activate bactericidal functions of Mtb-infected macrophages. Substantial evidence indicates that IFNγ can activate murine macrophages to limit Mtb growth [6]–[7] but the relative importance of this bactericidal mechanism and the cellular sources of IFNγ are unknown. Evidence for an CD4 T cell dependent, IFNγ independent mechanism of killing has been suggested by the finding that the frequency of Mtb-specific, IFNγ-producing cells following immunization does not correlate with protection against infection and that depletion of CD4 cells exacerbates Mtb infection in mice despite the ongoing expression of IFNγ [8], [9], [10], [11], [12].

In this report, we have assessed the requirement of IFNγ in protection by Mtb specific CD4 T cells. Using a model of adoptive transfer of Mtb specific effector CD4 T cells, we provide evidence for the surprising conclusion that IFNγ is not a required mediator of CD4 T cell defense to Mtb. In support of this finding is our discovery that key mediators of IFNγ- dependant immunity, inducible nitric oxide synthase and phagocyte oxidase, were not required for the early protective events mediated by adoptively transferred Th-1 skewed CD4 T cells. Although Th1-skewed cells were superior to Th2 or Th17-skewed cells in defense to Mtb, surprisingly, protection by Th1 –skewed cells was independent of the master regulator of Th1 differentiation, T-bet. Our results are contrary to a dominant role for IFNγ production by effector CD4 in Mtb protection, but strongly support a requirement for Th1 mediated immunity to Mtb.

## Results/Discussion

### IFN-gamma and iNOS independent control of *M. tuberculosis* infection

**Figure 1:**
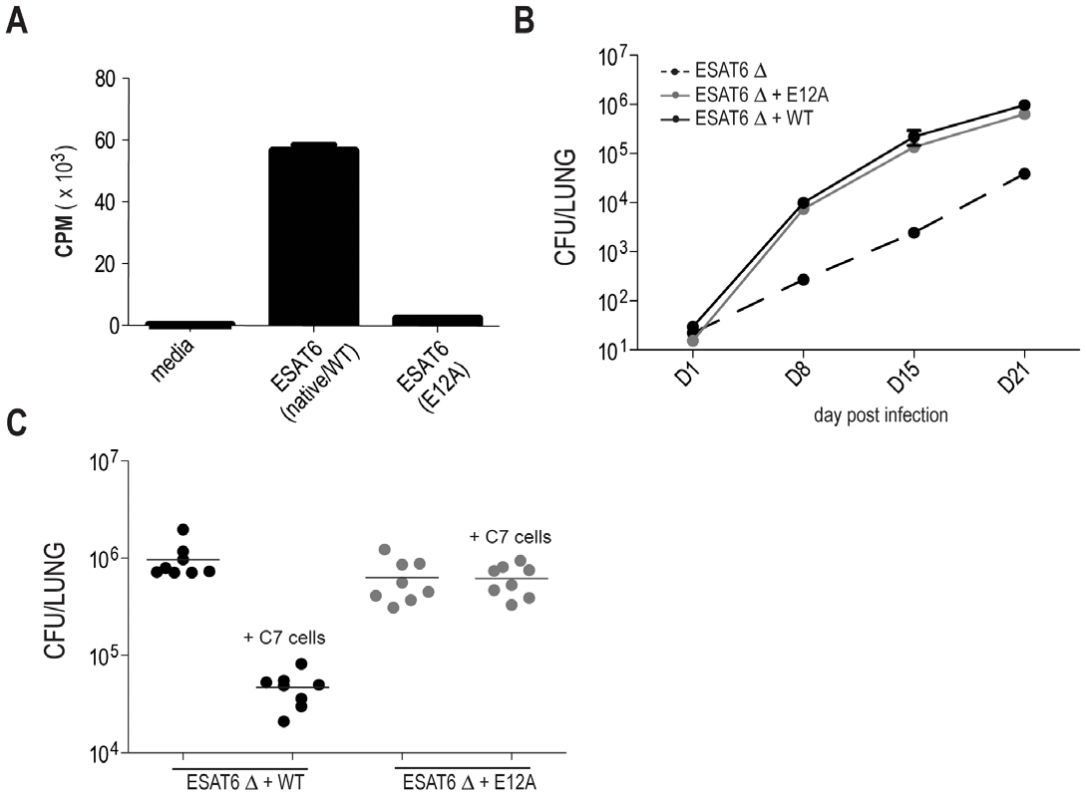
Mutation in amino acid 12 of ESAT6 prevents protection by C7 effector cells. (**A**) Naïve C7 CD4 T cells were cultured in the presence of irradiated APCs (T cell depleted splenocytes) and either native ESAT6 peptide (aa 1–20) or mutated ESAT6 peptide in which amino acid number 12 was changed from glutamic acid to alanine (E12A). Analysis of tritiated thymidine incorporation reveals C7 CD4 T cells do not respond to ESAT6 (E12A). (**B**) B6 mice were infected with the indicated strains of Mtb and CFUs determined. Each dot represents data from 4–10 mice. (**C**) Bacterial numbers from mice that received Th1-skewed C7 effector cells and were subsequently infected with Δ*esat6*+wt or Δ*esat6*+E12A. Bacterial numbers were determined 21 days post infection.

To investigate the contribution of IFNγ production by Mtb-specific CD4 T cells during infection, we compared the ability of WT and IFNγ deficient, Mtb-specific CD4 T cells derived from the C7 TCR transgenic mouse [13] , to protect mice from infection. Naive C7 CD4 T cells, specific for the immunodominant Mtb antigen ESAT-6 in the context of the IA^b^ MHC class II molecule, were activated in vitro under Th1-skewing conditions [13] and transferred into WT mice. To be certain that protection mediated by C7 CD4 T cells is antigen specific, we generated a strain of *M. tuberculosis* in which a key TCR contact residue in the ESAT-6 epitope (E12) was mutated to alanine to abolish C7 recognition (Figure 1A). *M. tuberculosis* ESAT6-E12A was fully virulent, but was not affected by Th1-differentiated C7 cells, whereas wild type *M. tuberculosis* titers were reduced by approximately 50 fold (Figure 1B and C).

**Figure 2:**
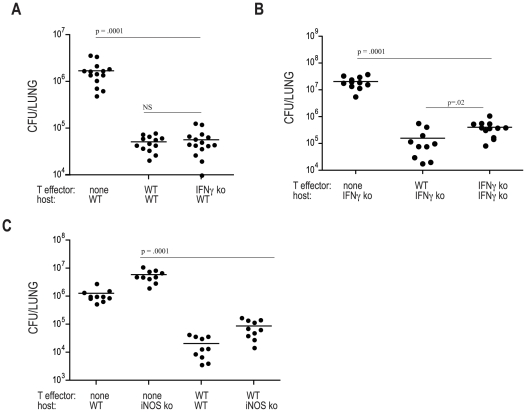
IFN-gamma and iNOS independent control of *M. tuberculosis* infection. ESAT-6 specific Th1-skewed CD4 effector cells were transferred into mice which were subsequently aerosol infected with ∼100 CFU of Mtb. Twenty-one days later lungs were harvested and plated to determine bacterial numbers. (**A/B**) *M. tuberculosis* numbers in the lungs of WT or IFNγ-deficient mice that either did not receive effector T cells, or received WT or IFNγ-deficient (ko) T cells. (**C**) Bacterial numbers harvested from either WT, or iNOS ko, mice that either did not receive effector T cells, or received Th1-skewed cells generated from WT ESAT-6 specific CD4 T cells. The data presented represents the combination of 2–3 experiments with 3–5 mice per group. Differences were calculated using unpaired Student's t test.

Next, we transferred ten million C7.WT or C7.IFNγ deficient Th1 effector cells into WT mice that were subsequently aerosol-infected with Mtb and, twenty-one days later, the number of bacteria in the lungs was determined. Surprisingly, both C7.WT and C7.IFNγ deficient cells provided similar levels of protection compared to animals that did not receive cells (Figure 2A), indicating that IFNγ production by adoptively transferred effector CD4 T cells is not required for protection when these cells are present at the time of infection. To examine whether IFNγ-independent restriction of Mtb growth is a specific property of C7 transgenic T cell populations or a general property of Mtb specific T cells, we generated bone marrow chimeric mice in which wild type recipient C57BL/6 mice received a 50∶50 mixture of bone marrow from Rag2-deficient and IFNγ-deficient donors. In these chimeric mice, all T cells are IFNγ-deficient, while Rag2-independent innate immune cells, such as NK cells, macrophages, monocytes and DCs are capable of producing IFNγ. Bone marrow chimeric mice with IFNγ-deficient T cells were similarly resistant to Mtb infection as mice receiving wild type T cells, supporting our conclusion that T cells can mediate protection without producing IFNg and that our results with transgenic T cells extend to native T cell populations (Figure S1). We also examined whether naïve C7 cells could limit *M. tuberculosis* growth and whether this effect is independent of IFN-γ. 10,000 naïve C7 cells significantly reduced *M. tuberculosis* bacterial load in the lung at 22 days (Figure S1B). IFNγ deficient T cells also significantly reduced bacterial loads and there was no significant difference in the ability of wild type and IFNγ deficient naïve cells to control *M. tuberculosis* growth (Figure S1B).

Because IFNγ is essential for effective immune control of Mtb, we speculated that IFNγ deficient C7 cells might recruit IFNγ-expressing host-derived cells (e.g. Natural Killer cells or endogenous CD4 or CD8 T cells) to sites of mycobacterial infection. In this way, host-derived IFNγ might activate the expression of mycobactericidal factors. To address this hypothesis, we tested that ability of adoptively transferred T cells to provide protection in mice lacking IFNγ. Remarkably, both WT and IFNγ-deficient C7 effector cells protected hosts lacking IFNγ, although in this setting IFNγ-deficient T cells were slightly but significantly less effective than WT C7 cells at limiting in vivo growth of Mtb. Nevertheless, compared to IFNγ deficient mice that did not receive T cells, animals that received C7 IFNγ deficient effectors had ∼30 fold reduction in bacterial numbers in the lungs at day 21 following infection (Figure 2B). This result demonstrates that CD4 T cells have a highly effective effector pathway to control Mtb that is completely independent of IFNγ.

During murine infection with Mtb, IFNγ signaling induces NOS2 (inducible nitric oxide synthase), leading to the generation of nitric oxide (NO) which can kill mycobacteria [14]. To determine whether adoptively transferred C7 T cells mediate protection by inducing NOS2, we transferred C7 T cells into NOS2 deficient mice. WT C7 effectors were effective at protecting both NOS2 and PHOX deficient mice from infection, resulting in ∼70 fold reduction in bacterial numbers in NOS2 or PHOX deficient C7-recipients compared to deficient mice that did not receive cells (Figure 2C) and Figure S2. NOS2 induction is a major IFNγ-dependent effector mechanism controlling defense against Mtb in mice, yet our results show that C7 T cells that produce IFNγ are similarly protective in WT and NOS2-deficient hosts. Taken together, our results demonstrate the existence of an IFNγ/NOS2-independent mechanism of CD4 T cell mediated killing of Mtb that is operative at the early time points examined in this study.

### Optimal control of *M. tuberculosis* growth can be independent of IFNγ and TNF production by effector T cells

**Figure 3:**
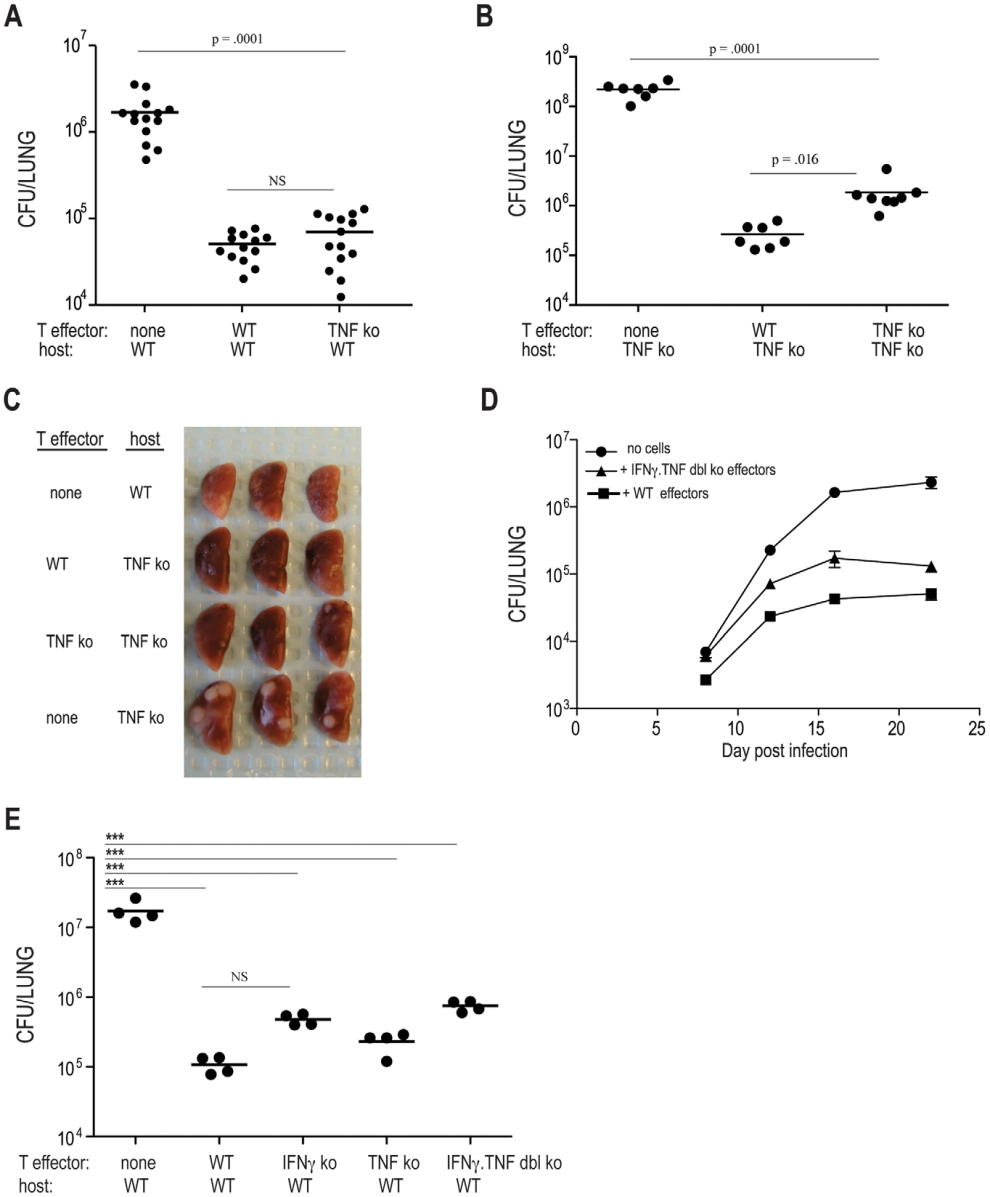
Optimal control of *M. tuberculosis* growth requires production of IFNγ and TNF by effector T cells. As in Figure 2, ten million (A–D) or one million (E) Th1-skewed ESAT-6 specific cells were transferred into the indicated hosts that were subsequently infected with Mtb. (**A/B**) Bacterial numbers in the lungs of WT or TNF deficient (ko) mice that either did not receive effector T cells, or received WT or TNF deficient effector cells. Bacterial numbers were determined 21 days post infection. The data are a combination of 2–3 experiments with 3–4 mice per group. (**C**) Pictures of lungs from 3 mice from the indicated experimental groups at day 21 post infection. (**D**) Bacterial numbers at the indicated times harvested from either WT mice that either did not receive cells, or received Th1-skewed WT or IFNγ.TNF dbl deficient cells. Each dot is the average of 7 mice from two independent experiments. Error bars mark SEM. (**E**) Bacterial numbers from mice receiving either no cells or 1 million C7 effector cells from the indicated donors. Differences were calculated using unpaired Student's t test (A, B, D), or calculated by one way ANOVA (E) ***p<0.0001.

Tumor necrosis factor (TNF) is another critical regulator of host defense that is secreted by Th1 CD4 T cells. The precise contribution of TNF to defense against Mtb infection is difficult to define since it has been implicated in lymphocyte recruitment, cell survival, and mycobacterial killing [3], [15], [16]. We next determined whether TNF deficient C7 cells could protect WT and TNF deficient mice from Mtb infection. The protection provided to recipient mice either by WT or TNF deficient C7 effector cells was comparable, demonstrating that TNF production by effector CD4 T cells is not required for protection against Mtb infection (Figure 3A). Similarly, TNF-deficient naïve C7 T cells provided the same level of protection as adoptively transferred wild-type C7 T cells (Figure S1B). Experiments using TNF deficient hosts showed that TNF deficient effectors reduced the number of bacteria by ∼100 fold, compared to TNF deficient hosts that did not receive C7 effectors (Figure 3B). Although adoptively transferred TNF-deficient C7 effectors were slightly less effective than WT C7 effectors at providing protection in TNF deficient recipients, TNF-deficient C7 effector T cells still provided a high degree of protection in TNF deficient recipients, an effect that could also be observed macroscopically in infected lungs, with TNF deficient hosts having large lesions that are diminished in size in TNF deficient recipients of either WT or TNF deficient C7 effectors (Figure 3C).

It is possible that the protection provided by IFNγ or TNF deficient C7 T cells might result from partial functional redundancy of these two cytokines. To test this idea, we generated C7 cells lacking both IFNγ and TNF (C7.IFNγ.TNF dbl deficient cells), and transferred these cells, following in vitro Th1 differentiation, into WT hosts and then infected with Mtb. Bacterial numbers were determined 8, 12, 16, and 22 days following aerosol infection and compared to control animals (recipients of WT effectors, or, animals that did not receive cells). The data in Figure 3Dγ demonstrate that when C7 T cells are unable to produce both TNF and IFN, their capacity to provide protection is modestly restricted. In comparison to mice that did not receive T cells and mice that received wild type C7 Th1 cells, double deficient T cells continued to provide ∼60% of maximal protection. TNF and IFNγ double deficient naïve C7 T cells also conferred protection against Mtb (Figure S1B). These results indicate that CD4 T cells, independent of both TNF and IFNγ, provide roughly 10 to 15 fold inhibition of Mtb growth.

We considered the possibility that the gamma/TNF independent pathway of antimycobacterial immunity demonstrated above might depend on cell dose. In the above experiments, the high cell dose (10 million) might allow a minor pathway of antimycobacterial immunity to substitute for the gamma/TNF pathway. To address this question, we repeated our experiments with one million transferred cells, which in our prior experience still provided substantial killing of *M. tuberculosis* [13]. One million transferred WT C7 cells reduced the bacterial load in the lungs of infected mice by 160 fold compared with animals that received no cells (Figure 3E). One million IFNγ deficient T cells retained substantial antimycobacterial effect, reducing bacterial loads by 36 fold compared to control animals (Figure 3E). Similarly, TNF deficient cells reduced bacterial loads by 74 fold. IFNγ/TNF deficient cells were somewhat impaired in their antimycobacterial effect, but still reduced bacterial loads by 23 fold. Taken together, these experiments indicate both IFNγ/TNF dependent and independent pathways of T cell mediated protection. The majority of protection conferred by the C7 cells is, however, IFNγ independent, even at lower T cell doses.

### Cytolysis via perforin and FAS are not required to control of *M. tuberculosis* infection

**Figure 4:**
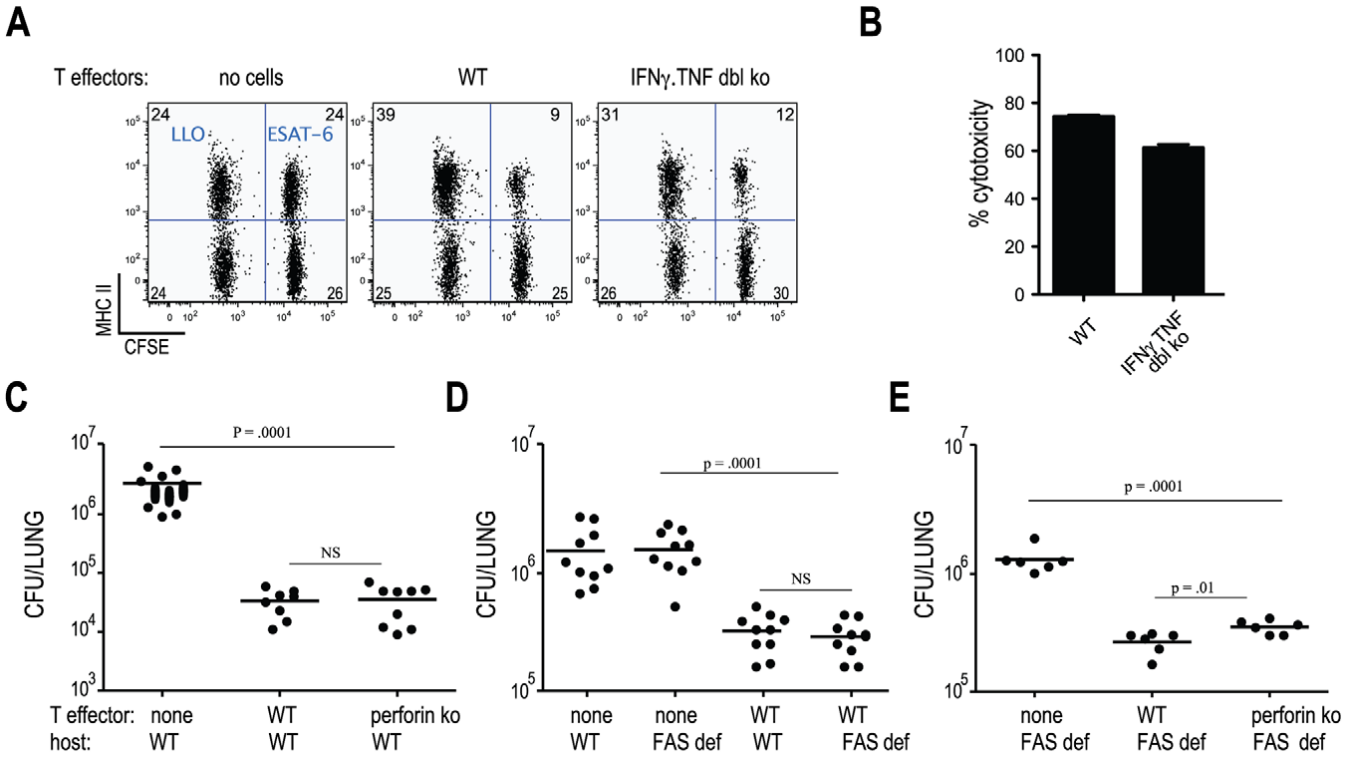
Cytolysis via perforin and FAS are not required for control of *M. tuberculosis* infection. (**A/B**) Th1-skewed ESAT-6 specific cells were transferred into WT mice (expressing CD45.2) that were subsequently transfused i.v. with equal numbers of CD45.1 CFSE^high^, ESAT-6 peptide-pulsed splenocytes, and CFSE^low^ control peptide-pulsed (Listeria lysin O, LLO) splenocytes. (**A**) In vivo cytotoxicity was assessed 16 hours later by flow cytometry. Dot plots of CFSE and MHC class II levels on CD45.1 splenocytes in recipient mice. The numbers represent frequencies of cells within each quadrant. (**B**) Cumulative data from two independent experiments from 3–4 mice per group. (**C–E**) Th1-skewed ESAT-6 specific cells from the indicated backgrounds were transferred into either WT or FAS deficient mice. Bacterial numbers were determined 21 days post infection. Experiments were performed 2 times with 3–5 mice per group. Statistical significance was calculated using the unpaired Student's t test.

MHC-class II restricted cytolytic activity has been observed following Mtb infection and has been suggested to contribute to protective immunity [17], [18]. Since Mtb-specific cells deficient in both IFNγ and TNF protected mice from infection, we investigated whether ESAT-6-specific CD4 T cell cytolytic activity contributed to in vivo protection. To determine whether C7 effector cells killed target cells presenting the ESAT-6 epitope in vivo, we transferred C7 WT or C7.IFNγ.TNF double deficient CD4 T cells into mice and 3 days later injected these mice with CFSE-labeled, ESAT-6-coated target cells. We detected approximately 60–80% specific lysis of ESAT-6-coated target cells in recipients of either C7.WT or C7.IFNγ.TNF double deficient effectors (Figure 4A and B). This high degree of cytolysis is similar to what was observed for endogenous populations of Mtb-specific CD4 T cells [17]. To determine the contribution of perforin in CD4 T cell-mediated protection against Mtb infection, we compared bacterial numbers in infected mice that did not receive cells to recipients of C7.WT or C7.perforin deficient, Th1-differentiated effector T cells. Figure 4C- demonstrates that perforin deficient T cells were able to restrict Mtb growth to the same extent as wild type T cells, indicating that perforinmediated cytolytic activity does not contribute to the T cell-mediated control of mycobacterial infection we observe.

Mice lacking functional FAS or FASL are more susceptible to Mtb infection than WT animals [19]. To determine if FAS mediated signaling contributes to C7 T cell-mediated protection of mice from Mtb infection, we modified our adoptive transfer protocol since C7.WT cells transferred before infection into FAS deficient hosts were undetectable 21 days following infection (data not shown). Instead of transferring C7 cells prior to infection, C7 effector cells were transferred 7 days following infection and bacterial numbers were measured 7 days later. The shorter experiment led to a similar recovery of C7 cells in WT and FAS deficient hosts (data not shown). Figure 4D( shows that deficiency in FAS signaling did not alter the ability of C7 effectors to protect against Mtb infection, since comparable bacterial numbers were cultured from WT and FAS deficient recipient mice. Elimination of both perforin and FAS had only a minor, albeit statistically significant effect on protection mediated by C7 cells Figure 4E). In addition, Perforin deficient C7 cells were also as capable as C7.WT cells at protecting iNOS deficient mice from infection (Figure S3). Taken together, these results demonstrate that early control of mycobacterial infection does not require CD4 T cells to kill Mtb-infected target cells by FAS or perforin-mediated cytolytic activity.

### Optimal protection to *M. tuberculosis* requires a Th-1 lineage population, yet is independent of T-bet

**Figure 5:**
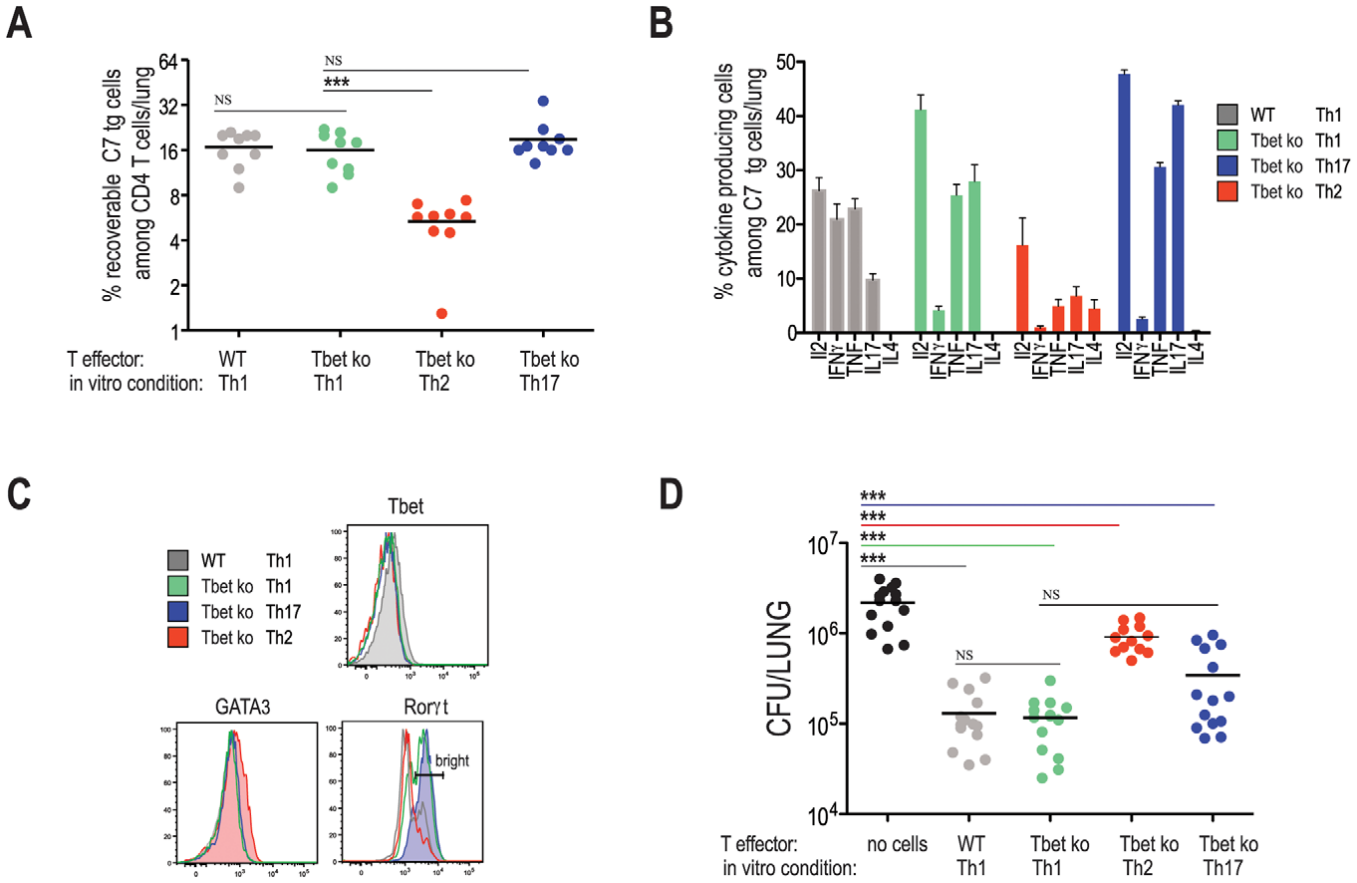
Optimal protection to *M. tuberculosis* requires a Th-1 lineage population, yet is independent of T-bet. ESAT-6 specific CD4 T cells from the indicated genetic backgrounds were activated for 4 days under Th1, Th2 or Th17 skewing conditions before transfer into the WT mice. Two days after transfer, mice were aerosol infected with ∼100 CFU of Mtb. Twenty-one days later lungs were harvested to determine frequencies and phenotypes of the transferred T cells and lungs were plated to determine bacterial numbers. (**A**) Frequencies of the transferred populations in the lungs of infected mice, as determined by flow cytometry. (**B**) Frequencies of IL2, IFNγ, TNF, IL17A, and IL4 producing cells among the transferred effector populations harvested from the lungs. (**C**) Histogram plots following intracellular staining for the indicated transcription factors. The gate marks the frequencies of RORγt bright cells. (**D**) Bacterial numbers in the lungs 21 days post infection. *** p<0.0001 calculated by one way ANOVA.

Because no single Th1-associated effector pathway was essential for T cell mediated protection against Mtb infection (i.e., IFNγ, TNF, perforin, and FAS), we asked whether protection is a general property of helper T cells regardless of their effector phenotype. To address this question, naïve C7 cells were differentiated into Th2 and Th17 cells and transferred into WT hosts prior to Mtb infection. C7 TCR tg mice were crossed to the T-bet deficient background to prevent in vivo conversion of Th2 and Th17 populations into cells with a Th1 profile (AMG and EGP unpublished data, and [20]). Twenty-one days following infection, lungs were harvested from infected animals and the frequencies and phenotypes of transferred populations were determined (Figure 5). While frequencies of Th1-skewed cells (on either a WT or T-bet deficient background), and Th17-skewed cells were similar, compared to these populations, the frequencies of Th2-skewed cells were reduced. Of note, adoptively transferred T cells, including Th2-skewed cells, inhibited the priming of host-derived endogenous populations of ESAT-6 specific cells, indicating that adoptively transferred cells were participating in the immune response (Figure S4). Since the degree of inhibition is directly correlated with the number of transferred effector cells (data not shown and [21]), we concluded from this result that recipient mice, at least at the time of endogenous T cell priming, harbored similar frequencies of adoptively transferred cells.

The cytokine and transcription factor profiles of adoptively transferred T cells correspond to the expected phenotypes based on the in vitro culturing conditions [22]. As expected, while WT Th1 cell populations contained IFNγ producing cells, all T-bet deficient cells examined (i.e., Th1, Th2, and Th17 skewed cells) did not produce detectable levels of IFNγ (Figure 5B). Th2-differentiated T-bet deficient C7 T cells expressed IL4 and GATA3, while Th17-skewed C7 cells expressed IL17 and RORγt (Figure 5B and C). Th1-skewed T-bet deficient C7 T cells also expressed IL17 and RORγt, albeit in lower proportions. Figure 5D demonstrates that Th2-skewed cells marginally protected mice from infection, demonstrating that protection is not a general feature of all effector CD4 T cells. Th1-skewed, T-bet-deficient C7 T cells protected mice as well as Th1-skewed, WT C7 T cells, corroborating our finding that IFNγ production by Mtb-specific CD4 T cells is unnecessary for early protection against Mtb infection. T-bet deficient C7 T cells differentiated under Th17 conditions provided protection, however [23], we consistently observed better protection by Th1-differentaited, T-bet deficient C7 T cells, despite seemingly similar cytokine and transcription factor profiles between these two populations (Figure 5B and C). Taken together, our results suggest that protection against Mtb infection is optimal when effector T cells are differentiated under Th1 conditions, however in a T-bet independent fashion.

IFNγ and TNF are central to host defense against Mtb infection in both mice and humans. However, because these cytokines have pleiotropic roles in T cell differentiation, cell trafficking, and macrophage activation, and are produced by a wide variety of immune cells, the exact mechanism(s) by which they confer protection has not been clearly defined. One predominant model is that Th1 effector T cells, which are known to produce both IFNγ and TNF, are the important in vivo sources of these cytokines and thereby activate macrophage mycobactericidal effector functions. However, there have been occasional reports in which protection against Mtb infection did not correlate with production of IFNγ by CD4 effector cells [5], [8], [9], [10], suggesting either that other cellular sources of these cytokines are important, or that IFNγ independent mechanisms of protection may exist. The data presented here provide strong support for a mycobactericidal effector function of CD4 T cells that is independent of both IFNγ and TNF. These results may indicate that the central role of these cytokines is to prime CD4 differentiation to the Th1 phenotype, after which other effector functions kill Mtb. When cells with a Th1 phenotype are supplied, IFNγ becomes dispensable. Our findings do not dispute the evidence that IFNγ plays an essential role in immunity to Mtb, rather they show that antigen experienced effector cells have mechanisms to control infection that do not rely on IFNγ mediated signals [24], [25]. When these cells are supplied at the time of infection, T cells can be highly effective at limiting Mtb growth. Our findings suggest that vaccination strategies that seek to maximize IFNγ producing CD4 T cells may miss an important effector mechanism of Th1 CD4 T cells that we demonstrate is highly effective at controlling Mtb growth in vivo. Further exploitation of this new pathway therefore holds promise for the design of vaccines to control Mtb infection.

## Materials and Methods

### Mice

This study was carried out in strict accordance with the recommendations in the Guide for the Care and Use of Laboratory Animals of the National Institutes of Health. The protocol was approved by the MSKCC Institutional Animal Care and Use Committee. No non-human primates were used in this research. C57BL/6J mice, iNOS ko (#002609), and FAS deficient mice (#000482) were purchased from Jackson Laboratory. C7.IFNγ ko, C7.TNF ko, C7.IFNγ.TNF dl ko, C7.perforin ko, C7.T-bet ko, IFNγ ko, TNF ko, and gp91 phox ko mice were maintained at the animal facility in the memorial Sloan-Kettering Research Animal Resource Center. The genotypes of the animals were confirmed by PCR analysis and phenotypic confirmation of the transgenic T cells was performed by intracellular staining (C7.IFNγ ko, C7.TNF ko, C7.IFNγ.TNF dbl ko, C7.T-bet ko). The Memorial Sloan-Kettering Institutional Animal Care and Use committee approved all animal procedures.

### Generation of effector T cells

4×10^6^ purified C7 TCR tg CD4+T cells were cultured with 12×10^6^ irradiated T cell–depleted splenocytes and 5 µg/ml of ESAT-6 1–20 peptide. At days 2 and 3 of culture, the cells were split 1∶2, and 50 U/ml IL-2 was added (R&D Systems). For Th1 cultures, 10 ng/ml IL-12, and 5 µg/ml of neutralizing anti–IL-4 antibody (R&D Systems) were added at day 0 of culture. For Th17 cultures, 10 µg/ml of anti-IFNγ and anti-IL-4 and 5 ng/ml of hTGF-β, 20 ng/ml of IL6 and 20 ng/ml of IL-23 were added at day 0 of culture. For Th2 cultures, 10 µg/ml of anti-IFNγ. and 20 ng/ml of IL4 were added to cultures.

### Aerosol infections with *M. tuberculosis* and generation of *M. tuberculosis* Erdman strains

Mice were infected at 8–10-wks of age with *M. tuber*culosis Erdman and plated as described [13] *M. tuberculosis* Erdman strain, Δ*esat6*::*hyg* (SSM6), has a deletion in the gene encoding ESAT-6 [26]. This strain was complemented with PMH406, which integrates at the chromosomal *attB* site, which uses the mop promoter to drive expression of CFP-10 and ESAT-6 [27]. Either wild type ESAT-6 or mutated ESAT-6 in which amino acid number 12 was changed from glutamic acid to alanine (E12A) were used to generate Δ*esat6* **::** *hyg attB*::pMH406 or Δ*esat6* **::** *hyg attB*::pMH406-ESAT6(E12A) .

### Statistical analysis

For comparison of means between two groups we performed the unpaired Student's t test in GraphPad Prism software. For experiments that involved more than three groups (e.g Figures 3E, 5A, 5D), we compared the groups using a one way ANOVA with a Bonferroni's multiple comparison test.

## Supporting Information

Figure S1 IFNγ independent control of Mtb infection by endogenous T cell populations or naïve C7 cells. **(A)** To generate bone marrow-chimeras, RAG ko mice were transplanted with BM from the indicated donors, for mixed BM- chimeras, 50% of the BM came from either WT or IFNγ ko mice. Animals were infected ∼8 weeks post transplant with Mtb. The data shows lung bacterial numbers 21 days post infection. **(B)** Naïve C7 cells have an IFN gamma independent pathway of antimycobacterial immunity. 10,000 naïve C7 cells of the indicated genotype were transferred on the day before infection and bacterial loads in the lungs of infected mice were determined 22 days after infection. * p<0.05; ** p<0.001 calculated by one way ANOVA.
(PDF)

Figure S2 C7 effector cells protect PHOX ko mice from Mtb infection. Bacterial numbers of either WT or PHOX ko mice that either did not receive cells or received WT Th1-skewed C7 effector cells. The data shows lung bacterial numbers 21 days post infection. Differences were compared using unpaired Student's t test.
(PDF)

Figure S3 C7.perforin ko effector cells protect iNOS ko mice from Mtb infection. Bacterial numbers 21 days post infection in iNOS ko mice that either did not receive effector cells or received either perforin ko or WT Th1 skewed effector cells. Differences were tested using unpaired Student's t test.
(PDF)

Figure S4 C7 effector T cells prevent activation of host-derived ESAT-6 specific cells. C7 CD4 T cells from the indicated genetic backgrounds were activated in vitro under Th1, Th2, or Th17-skewing conditions. These cells were transferred into B6 mice that were subsequently infected with Mtb. Twenty-one days later, the frequencies of host-derived ESAT-6 specific cells in the lungs were determined by intracellular cytokine staining following ESAT-6 stimulation. (**A)** Flow cytometry plots gated on host-derived CD4 T cells, demonstrates that host-derived ESAT-6 specific cells (IFNg + TNF +) are undetectable in animals that received Th1, Th2, and Th17-skewed cells. **(B)** Analysis from 5–10 mice per experimental group.
(PDF)
