# Supplementary figures and images for: A Gamma Interferon Independent Mechanism of CD4 T Cell Mediated Control of M. tuberculosis Infection in vivo
Source: PLoS Pathog. 2011 May 19;7(5):e1002052. doi: 10.1371/journal.ppat.1002052 (PMC3098235; doi:10.1371/journal.ppat.1002052)

## Supporting Figure 1

**A**

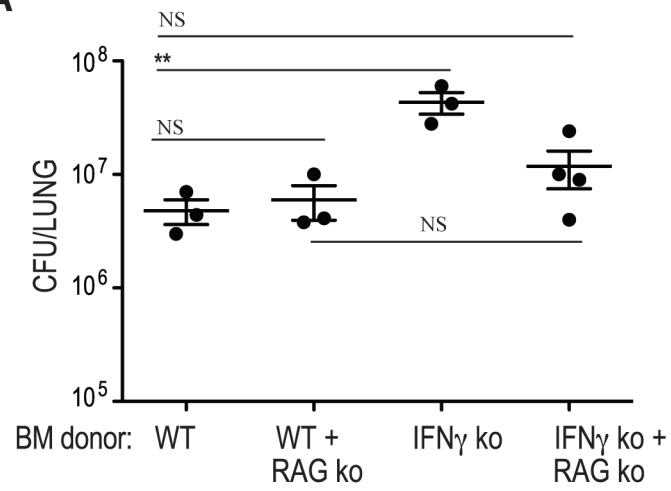

**B**

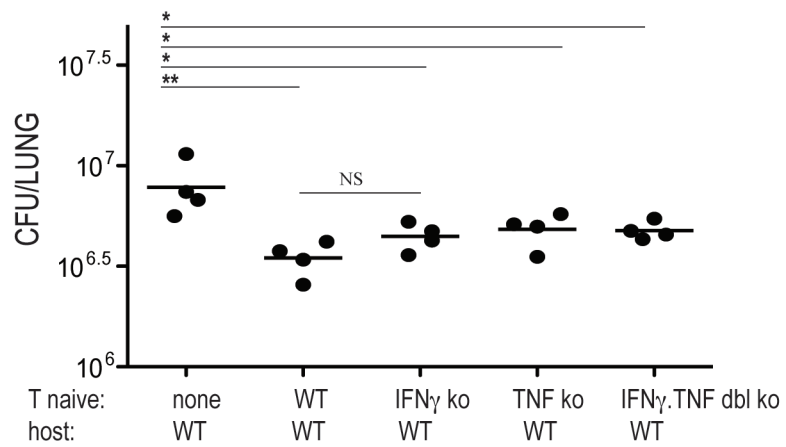

Supplement: Figure S1 — IFNγ independent control of Mtb infection by endogenous T cell populations or naïve C7 cells. (A) To generate bone marrow-chimeras, RAG ko mice were transplanted with BM from the indicated donors, for mixed BM- chimeras, 50% of the BM came from either WT or IFNγ ko mice. Animals were infected ∼8 weeks post transplant with Mtb. The data shows lung bacterial numbers 21 days post infection. (B) Naïve C7 cells have an IFN gamma independent pathway of antimycobacterial immunity. 10,000 naïve C7 cells of the indicated genotype were transferred on the day before infection and bacterial loads in the lungs of infected mice were determined 22 days after infection. * p<0.05; ** p<0.001 calculated by one way ANOVA. (PDF) [file ppat.1002052.s001.pdf]

## Supporting Figure 2

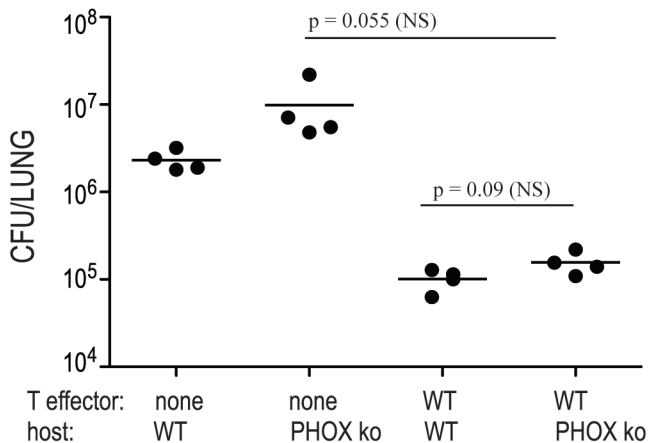

Supplement: Figure S2 — C7 effector cells protect PHOX ko mice from Mtb infection. Bacterial numbers of either WT or PHOX ko mice that either did not receive cells or received WT Th1-skewed C7 effector cells. The data shows lung bacterial numbers 21 days post infection. Differences were compared using unpaired Student's t test. (PDF) [file ppat.1002052.s002.pdf]

### Supporting Figure 3

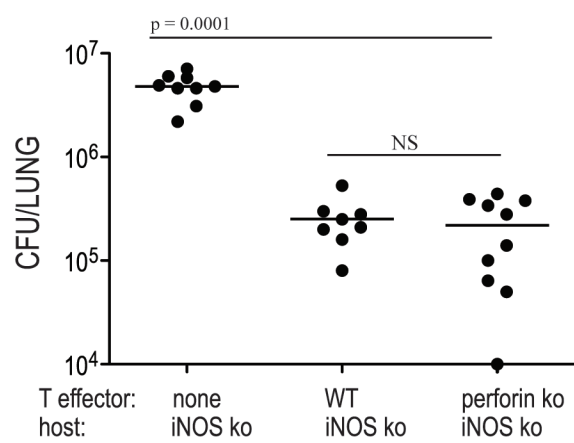

Supplement: Figure S3 — C7.perforin ko effector cells protect iNOS ko mice from Mtb infection. Bacterial numbers 21 days post infection in iNOS ko mice that either did not receive effector cells or received either perforin ko or WT Th1 skewed effector cells. Differences were tested using unpaired Student's t test. (PDF) [file ppat.1002052.s003.pdf]

## Supporting Figure 4

**A**

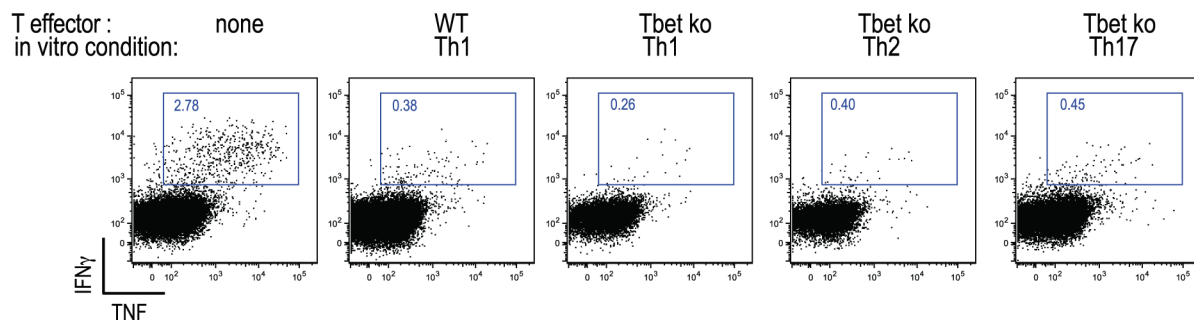

**B**

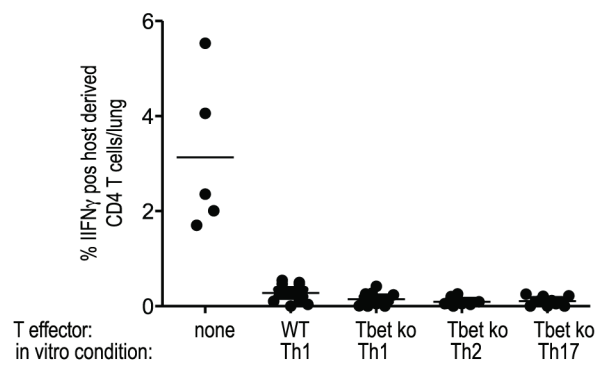

Supplement: Figure S4 — C7 effector T cells prevent activation of host-derived ESAT-6 specific cells. C7 CD4 T cells from the indicated genetic backgrounds were activated in vitro under Th1, Th2, or Th17-skewing conditions. These cells were transferred into B6 mice that were subsequently infected with Mtb. Twenty-one days later, the frequencies of host-derived ESAT-6 specific cells in the lungs were determined by intracellular cytokine staining following ESAT-6 stimulation. (A) Flow cytometry plots gated on host-derived CD4 T cells, demonstrates that host-derived ESAT-6 specific cells (IFNg + TNF +) are undetectable in animals that received Th1, Th2, and Th17-skewed cells. (B) Analysis from 5–10 mice per experimental group. (PDF) [file ppat.1002052.s004.pdf]
